# Prevention of Stress-Induced Depressive-like Behavior by Saffron Extract Is Associated with Modulation of Kynurenine Pathway and Monoamine Neurotransmission

**DOI:** 10.3390/pharmaceutics13122155

**Published:** 2021-12-14

**Authors:** Camille Monchaux De Oliveira, Véronique De Smedt-Peyrusse, Jennifer Morael, Sylvie Vancassel, Lucile Capuron, David Gaudout, Line Pourtau, Nathalie Castanon

**Affiliations:** 1 INRAE, Nutrition and Integrative Neurobiology (NutriNeuro), UMR 1286, 33076 Bordeaux, France; c.monchaux@activinside.com (C.M.D.O.); veronique.peyrusse@yahoo.fr (V.D.S.-P.); jennifer-nathalie.morael@etu.u-bordeaux.fr (J.M.); sylvie.vancassel@inrae.fr (S.V.); lucile.capuron@inrae.fr (L.C.); 2 Department of Life Science and Health, Nutrition and Integrative Neurobiology (NutriNeuro), Bordeaux University, UMR 1286, 33076 Bordeaux, France; 3 Activ’Inside, 33750 Beychac-et-Caillau, France; d.gaudout@activinside.com (D.G.); l.pourtau@activinside.com (L.P.)

**Keywords:** depressive disorders, nutritional interventions, saffron extract, Safr’Inside™, acute restraint stress, depressive-like behavior, inflammation, monoaminergic systems, kynurenine pathway

## Abstract

Depressive disorders are a major public health concern. Despite currently available treatment options, their prevalence steadily increases, and a high rate of therapeutic failure is often reported, together with important antidepressant-related side effects. This highlights the need to improve existing therapeutic strategies, including by using nutritional interventions. In that context, saffron recently received particular attention for its beneficial effects on mood, although the underlying mechanisms are poorly understood. This study investigated in mice the impact of a saffron extract (Safr’Inside™; 6.25 mg/kg, *per os*) on acute restraint stress (ARS)-induced depressive-like behavior and related neurobiological alterations, by focusing on hypothalamic–pituitary–adrenal axis, inflammation-related metabolic pathways, and monoaminergic systems, all known to be altered by stress and involved in depressive disorder pathophysiology. When given before stress onset, Safr’Inside administration attenuated ARS-induced depressive-like behavior in the forced swim test. Importantly, it concomitantly reversed several stress-induced monoamine dysregulations and modulated the expression of key enzymes of the kynurenine pathway, likely reducing kynurenine-related neurotoxicity. These results show that saffron pretreatment prevents the development of stress-induced depressive symptoms and improves our understanding about the underlying mechanisms, which is a central issue to validate the therapeutic relevance of nutritional interventions with saffron in depressed patients.

## 1. Introduction

Depressive disorders are among the most common and debilitating psychiatric illnesses, affecting over 322 million people worldwide [1]. To make matters worse, their prevalence is constantly rising despite currently available treatment options, thus complicating patient management and care. Highly prevalent in people afflicted with chronic inflammatory conditions [2,3,4] or exposed to stressful life events [5,6,7], depression is often characterized by chronic relapse. Moreover, a significant proportion of patients does not respond to conventional antidepressants (ADs), while still developing aversive side effects [8]. These major health concerns emphasize the need to expand treatment options by identifying new therapeutic strategies able to effectively target the complex pathophysiological mechanisms of depression.

As the majority of studies carried out to decipher the neurobiological underpinnings of depression started by highlighting the role of brain monoamine deficiency, most conventional ADs primarily aim to increase the synaptic availability of neurotransmitters, mainly serotonin (5-HT), but also dopamine (DA) and noradrenaline (NA), by specifically acting on their receptors, transporters, and/or catabolic enzymes [9,10,11]. However, it is now known that many of these medications, particularly those inhibiting monoamine reuptake, can also act by targeting other pathophysiological mechanisms of depression [12], including dysregulation of the hypothalamic–pituitary–adrenal (HPA) axis, which is classically characterized by increased cortisol levels and the desensitization of glucocorticoid receptors (GR) resulting in impaired glucocorticoid negative feedback [5,13,14,15] and alterations of hippocampal neuroplasticity, reflected in decreased brain derived neurotrophic factor (BDNF) levels [16,17,18].

More recently, other theories about the etiology of depression have emerged, notably related to the involvement of inflammatory processes [19]. In this context, growing attention has been paid to the critical role of two metabolic pathways, the kynurenine (KYN) and tetrahydrobiopterin (BH4) pathways, whose alteration in inflammatory conditions ultimately impairs monoaminergic neurotransmission, while inducing depressive symptoms [20,21]. Upon inflammatory activation, the indoleamine 2,3-dioxygenase (IDO) degrades the 5-HT precursor tryptophan into KYN, at the expense of 5-HT. Concurrently, the inflammation-driven activation of downstream enzymes of the KYN pathway promotes glutamate-related neurotoxicity through the synthesis of several KYN neurotoxic derivatives [18,22]. Inflammatory cytokines also dysregulate the BH4 pathway, particularly by acting on the GTP-cyclohydroxylase-1 (GTPCH1) and in turn favoring the production of toxic derivatives at the cost of BH4. Since BH4 is an essential cofactor for monoamine synthesis including DA, its disruption ultimately impairs DA neurotransmission likely to contribute to depressive symptoms [22]. Accordingly, the KYN and BH4 pathways have been proposed as potential therapeutic targets for the treatment of depressive symptoms occurring notably in contexts of inflammation [22,23].

Based on this knowledge, research aiming to identify new treatment options for depression has been mainly directed towards the possibility of improving if not all, at least several of the neurobiological alterations just mentioned. For this purpose, and keeping in mind the need to concomitantly reduce side effects accompanying pharmacological treatments, special interest has been recently paid to alternative therapeutic strategies. They particularly include nutritional interventions using essential nutrients or bioactive plant extracts with potential neuromodulatory and/or immunomodulatory properties [24,25]. In that context, saffron, a spice extracted from Crocus sativus L and used for centuries for its positive impact on health, appears as a promising candidate [26,27]. Over the last decades, saffron bioactive compounds have received more and more attention for their multiple valuable therapeutic properties, including antioxidant, anti-inflammatory, anxiolytic, or antidepressant properties [28,29,30,31]. Interestingly, compelling clinical studies have already shown that saffron administration improves mood in patients suffering from mild to major depression [26,27,32,33]. Several preclinical studies support these findings by reporting a reduction in depressive-like behaviors following saffron extract administration [34,35,36,37]. Nicely extending these findings, we recently demonstrated in naive mice that this behavioral improvement is associated with modulation of monoaminergic neurotransmission [38]. Beyond this neuromodulatory impact [38,39], saffron was also found to modulate the redox and inflammatory status [29,40], as well as HPA axis activity [41,42], although it has been suggested that it may preferentially interfere with the HPA axis under stressful rather than basal conditions [43,44]. However, the contribution of these different mechanisms to the behavioral effects of saffron remains to be confirmed, particularly in stressful conditions that play an essential role in the etiology of depression [5,6,7].

In order to address this issue, the present study aimed to assess the effects of an oral administration of Safr’Inside, a standardized saffron extract, on stress-induced depressive-like behavior and related neurobiological alterations in mice. Saffron was provided either before or just after stress exposure, in order to dissociate the potential preventing effect from a treatment effect. The acute restraint stress (ARS) paradigm was chosen because it elicits depressive-like behaviors, together with the dysregulation of most of the main neurobiological systems underlying their induction and suspected to be directly or indirectly targeted by conventional ADs [45,46,47]. This study shows that only Safr’Inside pretreatment prevents stress-induced depressive-like behavior and highlights the improvement of inflammatory processes and monoamine neurotransmission as potential underlying mechanisms.

## 2. Materials and Methods

### 2.1. Animals and Treatment

Eight-week old male C57BL/6J mice were obtained from Janvier labs (Le Genest-Saint-Isle, France). Upon arrival, they were randomly allocated to the different experimental groups and housed collectively (7–8 mice/cage) in an enriched (cardboard rodent homes and cotton nestlets) and controlled environment (22 ± 2 °C, 40% of humidity), with a 12 h/12 h light/dark cycle (light on at 7:30 a.m.) and free access to water and food (Standard Rodent Diet A04, SAFE, Augy, France). All animal procedures were conducted in strict compliance with the European Union recommendations (2010/63/EU) and were approved by the local ethical committee (approval ID A16873). Maximal efforts were made to reduce the suffering and number of animals used.

On the day of the test, a freshly prepared solution of saffron extract and its vehicle (water) were orally administered using a mouse-adapted feeding probe (ECIMED 1.33 × 30 mm). The saffron extract (Safr’Inside™; Activ’Inside, Beychac-et-Caillau, France) was a standardized extract obtained according to the patent FR 3054443 and containing more than 25 active compounds, including crocins (>3%), safranal (>0.2%), picrocrocin derivatives (>1%), and kaempferol derivatives (>0.1%), as measured by the U-HPLC method. The dose of Safr’Inside™ used (6.25 mg/kg *per os*), as well as its route (gavage) and volume of injection (10 mL/kg), were chosen based on previous studies [38,48]. In order to minimize stress reaction, mice were handled and habituated to the gavage procedure for several days before the test.

### 2.2. Experimental Design

**Figure 1:**
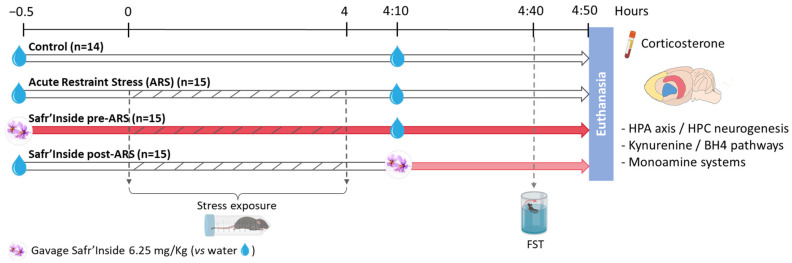
Schematic view of the experimental design. ARS: Acute Restraint Stress; FST: Forced Swim Test; HPA axis: Hypothalamo-Pituitary-Adrenal axis; HPC: Hippocampus; BH4: Tetrahydrobiopterin.

The experimental design is summarized in Figure 1. Control mice (*n* = 14 mice) were unstressed and received only water. Stressed mice were submitted to a 4-h acute restraint stress (ARS) and randomly distributed into 3 groups (*n* = 15/group) administered with Safr’Inside™ (30 min before the stress onset or 10 min after its end), or with water (at the two timepoints) for mice non-treated with saffron. The ARS procedure was essentially conducted as previously described [45]. Briefly, stressed mice were immobilized using polypropylene conical tubes (29.1 mm in diameter × 114.4 mm long) pierced with multiple holes to allow breathing and to limit the rise in body temperature. Four hours later, they were removed from the restraint tubes and put back to their respective home cage. Control mice stayed in their home cage during the entire stress procedure. All mice were tested in the forced swim test (FST) 30 min after the last administration of saffron extract or water and euthanized immediately after the behavioral test.

### 2.3. Behavioral Measures

Behavioral characterization was carried out during the light phase in a devoted sound-proof room equipped with a recording device that allows the behaviors to be analysed later by a trained observer blind to experimental conditions, using an ethological software (“The Observer XT 15”, Noldus, The Netherlands). The FST was used here to measure depressive-like behaviors. This well-validated test is routinely employed in pharmacological studies to screen drugs based on their possible ability to reduce these behaviors [49].

As previously described [38,50], mice were placed individually in a cylindrical glass tank (diameter: 16 cm; height: 31 cm) containing warm water (25 °C +/−1 °C) for 6 min during which the duration of swimming, climbing, and immobility was measured. Water was changed between each session. Increased immobility time is believed to reflect a state of helplessness that is reduced by conventional ADs. To further evaluate the impact of saffron extract on depressive-like behavior, we also determined within each experimental group the proportion of mice that displayed longer immobility than an immobility threshold, which was defined as the average percentage of time spent immobile by the control group [38].

### 2.4. Tissues Sampling

At the end of the FST, mice were euthanized with terminal pentobarbital/lidocaine anesthesia (300/30 mg/kg, intraperitoneally). Blood samples were immediately collected from the heart into tubes coated with an anticoagulant (EDTA 10%) and centrifuged (2000× *g*) for 20 min at 4 °C. Supernatants containing plasma fraction were next aliquoted and stored at −80 °C until corticosterone content was assayed. A transcardiac perfusion with chilled PBS 1X (2 min, 10 mL/min) was then rapidly performed in order to clean tissues from all traces of blood. Brains were extracted from the skulls and carefully dissected to hemilaterally collect structures of interest, i.e., the frontal cortex (FCx), striatum (STR), and hippocampus (HPC), which were immediately placed in sterile tubes, dry ice frozen, and stored at −80 °C for further analysis.

### 2.5. Enzyme Immunoassays (EIA)

The Corticosterone-HS kit (ImmunoDiagnostic System, Pouilly, France) was used to measure plasma corticosterone levels following the manufacturer’s instructions. All samples were diluted 10× and run in duplicate. The absorbance at 450 nm was measured by spectrophotometry (Victor3V, PerkinElmer, Villebon-sur-Yvette, France). Corticosterone concentrations (expressed in ng/mL) were calculated according to the standard range provided by the supplier.

### 2.6. High Performance Liquid Chromatography Coupled to Electrochemical Detection (HPLC-EC)

Concentrations of monoamines (DA, 5-HT) and their metabolites (dihydroxyphenylacetic acid (DOPAC), homovanillic acid (HVA), and 5-hydroxyindoleacetic acid (5-HIAA)) were measured in the FCx, STR, and HPC by HPLC-EC, essentially as previously described [38]. Briefly, 600 µL of extraction buffer were added to the different brain structures, which were then homogenized in a TissuLyser system (3 × 1 min at 30 Hz, 4 °C; Qiagen, Courtaboeuf, France). After 20 min of centrifugation (16,000× *g*, 4 °C), the supernatant containing the analytes to be measured was collected and divided into two aliquots. The first was immediately frozen at −80 °C for protein analysis by Western blotting (WB), while the second was centrifuged further for 2 min in filter tubes (1600× *g*, 4 °C) before being stored at −80 °C until use for HPLC-EC. For this purpose, 20 µL of each sample were injected into a high-performance liquid chromatograph equipped with an electrochemical detector coupled to a Chromeleon integration 6.8 software (Dionex, Sunnyvale, CA, USA), which allows the detection of the different analytes based on their respective retention time. Final concentrations were calculated against external standards, which were injected twice daily, and expressed per g of fresh tissue.

### 2.7. Real-Time Quantitative PCR (RT-qPCR)

RT-qPCR was used to assess the expression of the different genes of interest in selected brain areas depending on their relevance for the systems considered. Thus, dopaminergic markers, including DA receptors (DRD1 and DRD2), transporter (DAT), and degradation enzyme catechol-O-methyltransferase (COMT), were measured in the STR and FCx, two major brain areas of the dopaminergic system, while 5-HT receptors (5-HTR1a and 5-HTR1b) and transporter (SERT) were assessed in the FCx and HPC because of their richness in 5-HT synapses [51,52]. BDNF expression was measured in the HPC since it is a crucial brain area for neurogenesis [16]. Lastly, glucocorticoid receptors (GR), monoamine oxidase degradation enzymes (MAO-A and MAO-B), as well as key enzymes of the KYN pathway (indoleamine 2,3-dioxygenase (IDO), kynurenine 3-monooxygenase (KMO), kynurenine aminotransferase (KAT), kynureninase (KYNU), and 3-hydroxyanthranilate 3,4-dioxygenase (HAAO)) and BH4 pathway (GTP-cyclohydrolase I (GTPCH1), 6-pyruvoyl tetrahydropterin (PTS) and sepiapterin reductase (SPR)) were measured in the FCx, STR, and HPC.

RT-qPCR was performed as previously described [38]. Briefly, total RNAs were extracted from half brain structures using Trizol (Invitrogen, Life Technologies, Villebon-sur-Yvette, France) and reverse-transcribed into complementary DNA using Superscript III (Invitrogen, Life Technologies, Villebon-sur-Yvette, France). For the amplification, 2 µL of cDNA at 20 µg/µL were run in duplicate with Taqman LightCycler^®^ 480 Probes Master mix (Roche Diagnostics, Meylan, France) and appropriate FAM-labeled Taqman primers (ThermoFisher Scientific, Waltham, MA, USA). Fluorescence was measured by a Light cycler 480 II system (Roche Diagnostics, Meylan, France). Results were normalized using Beta-2-Microglobulin (B2M) as a house-keeping gene and expressed as relative expression compared to the control group. All primer references are given in Supplementary Table S1.

### 2.8. Western Blotting (WB)

Protein levels of DA and 5-HT receptors and transporters were assessed by WB in the same brain areas as gene expression. In order to optimize these measures while avoiding the management of many samples at the same time, which can unspecifically increase the interindividual variability, they were performed in 2 steps. The first aimed to compare Safr’Inside-treated stressed mice with their untreated counterparts. The second step, only carried out for proteins differentially expressed between these two groups, was then dedicated to compare the control and ARS groups, to determine if saffron selectively acts on stress-induced protein level alterations or independently from stress.

Total protein concentration was determined in each sample using the MicroBC assay protein quantitation kit following the manufacturer’s protocol (UP40840A, Interchim, Montluçon, France). During thawing, samples were treated with protease inhibitors (Complet ultra tablet, Roche Diagnostics, Meylan, France) and buffered with NaOH 1 mol/L to adjust the acidic pH of the extraction buffer. Then, equal quantities of proteins were electrophoresed on 12% sodium polyacrylamide-dodecyl sulfate gel with a 4% stacking gel and transferred to nitrocellulose membranes (Amersham 300 × 4 mm, UGAP, Champs-sur-Marne, France) as previously described [53]. Membranes were saturated 1 h in 5% milk (Regilait, UGAP, Champs-sur-Marne, France) or 5% BSA (P06-139,1100, Dutscher, Montsaunès, France) in Tris-Buffered Saline (TBS) and Tween 0.1%, then incubated with different primary antibodies overnight at 4 °C: anti-DRD1 (D2944; Sigma, Molsheim, France), anti-DRD2, anti-DAT (AB5084P, AB2231 respectively; Millipore, Molsheim, France), anti-SERT, anti-5-HTR1a (ab172884, ab85615 respectively; Abcam, Cambridge, UK) and anti-GAPDH (glyceraldehyde 3-phosphate dehydrogenase) as maintenance protein (5174S, rabbit; Cell Signaling, Leiden, The Netherlands). The amount of proteins and antibody concentrations used for each target are detailed in Supplementary Table S2. After washing in TBS-Tween, all membranes were incubated for 1 h at room temperature with appropriate secondary antibodies (1/5000) conjugated to donkey horseradish peroxidase (HRP) (Interchim, Montluçon, France). They were then washed, incubated for 5 min with peroxidase revealing solution (SuperSignal West Dura, ThermoFisher, Waltham, MA, USA), and revealed using ChemiDoc MP detection system (Biorad, Hercules, CA, USA) to measure chemiluminescence. Signals intensities were quantified using Image Lab 5.2.1 software (Biorad, Hercules, CA, USA) and proteins of interest were normalized to the house-keeping protein GAPDH.

### 2.9. Statistical Analyses

Statistical analyses were performed using Statistica 6 software (StatSoft, Tulsa, OK, USA), and possible outliers were identified with Graphpad Outlier Calculator [54] to be removed from the data. First, normality was assessed using the Shapiro–Wilk test. Parametric statistics with groups as between-factor were used when distribution was normal using one-way ANOVA, followed by Fisher LSD post-hoc test when necessary. For non-normal distribution, statistical validity was assessed with non-parametric test (Kruskal–Wallis H test followed by multiple comparison of ranks when appropriate). WB were analyzed using an unpaired t-test or Mann–Whitney U test depending on the normality. Immobility index was analysed with the Fisher’s exact test on contingency tables. The statistical level of significance was set at *p* ≤ 0.05. All data are presented as means ± SEM.

## 3. Results

### 3.1. Safr’Inside Administration Does Not Modify ARS-Induced Weight Loss

**Figure 2:**
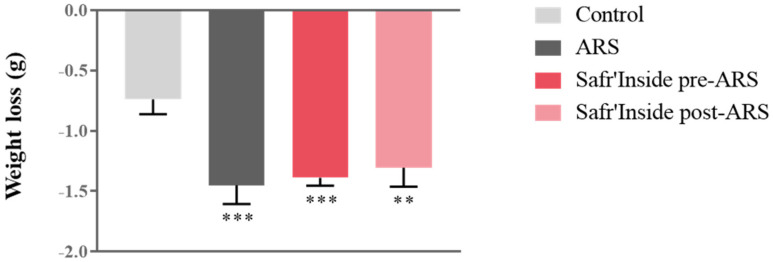
Effect of acute restraint stress (ARS) and oral administration of Safr’Inside™ (6.25 mg/kg) on body weight. Weight loss (g) during the 4 h of stress; Results are shown as mean ± SEM. Control: *n* = 14; ARS: *n* = 15; Safr’Inside pre-ARS: *n* = 15; Safr’Inside post-ARS: *n* = 15. ** *p* ≤ 0.01; *** *p* ≤ 0.001 vs. Control.

We first measured body weight changes as a classical physiological index of stress impact. Although all groups displayed similar body weight before stress exposure, we observed differences in weight loss at the end of the procedure (F_(1,55)_ = 348; *p* ≤ 0.001). Indeed, the ARS procedure significantly induces weight loss in all stressed mice compared to controls, regardless of pre/post-ARS Safr’Inside treatment, as revealed by post hoc analyses (Figure 2).

### 3.2. Safr’Inside Administration Reduces ARS-Induced Depressive-like Behavior Only When Given before Stress Exposure

**Figure 3:**
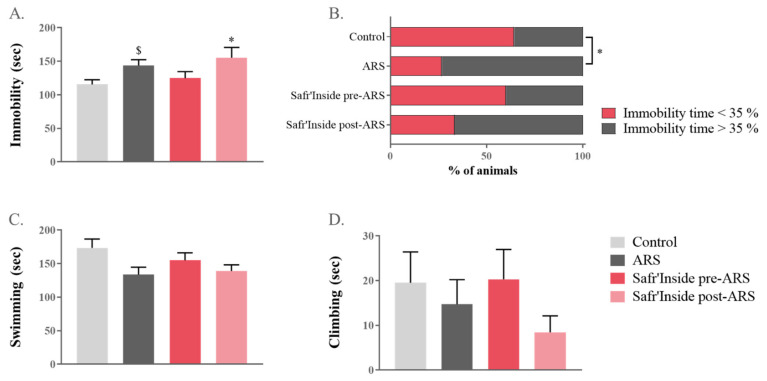
Effect of acute restraint stress (ARS) and oral administration of Safr’Inside™ (6.25 mg/kg) on depressive-like behavior measured in the forced swim test (FST). (**A**) Immobility time (sec); (**B**) Index of Immobility; (**C**) Swimming time (sec) and (**D**) Climbing time. FST has been conducted 30 min after the last gavage. Results are shown as mean ± SEM. Control: *n* = 14; ARS: *n* = 15; Safr’Inside pre-ARS: *n* = 15; Safr’Inside post-ARS: *n* = 15. * *p* ≤ 0.05 vs. Control (post hoc test), $ *p* ≤ 0.05 vs. Control (*t*-test).

The impact of pre-stress and post-stress Safr’Inside administration on ARS-induced depressive-like behavior was assessed in a classical rodent test of depression, the FST [49]. A one-way ANOVA revealed a significant overall difference between groups in the immobility time (F_(1,55)_ = 620; *p* ≤ 0.05; Figure 3A). Additional post hoc analyses showed that this parameter was differentially increased by ARS depending on treatment conditions. Specifically, mice treated after stress exposure are significantly more immobile than controls (Safr’Inside post-ARS vs. Control: *p* ≤ 0.05), while it is not the case for those receiving a pre-administration of saffron. Although the difference between untreated stressed mice and controls does not reach significance when analysed with a global post-hoc test (*p* = 0.07), a direct group-by-group comparison revealed a significant effect of ARS (ARS vs. Control: t_(1,14)_ = 2.49; *p* ≤ 0.05). Importantly, this was confirmed by the immobility index that is similar in the pretreated and control groups (Figure 3B), but different from that of untreated stressed group (ARS vs. Control *p* ≤ 0.05; Safr’Inside pre-ARS vs. ARS: *p* = 0.06). This index reflects, for each experimental group, the proportion of mice spending more time immobile than the average percentage of time spent immobile by control mice (35.7%). Interestingly, this proportion is drastically increased in untreated-stressed mice (73.3%) and mice treated after stress (66.7%), while it remains very close to that of controls in the saffron pretreated group only (40.0%). Lastly, ARS also tends to decrease swimming time (one-way ANOVA F_(1,55)_ = 716; *p* = 0.06; Figure 3C), while climbing time is unchanged and very short regardless of the group (Figure 3D).

### 3.3. Safr’Inside Administration Only Slightly Changes HPA Axis Function and Related Neurobiological Targets

**Figure 4:**
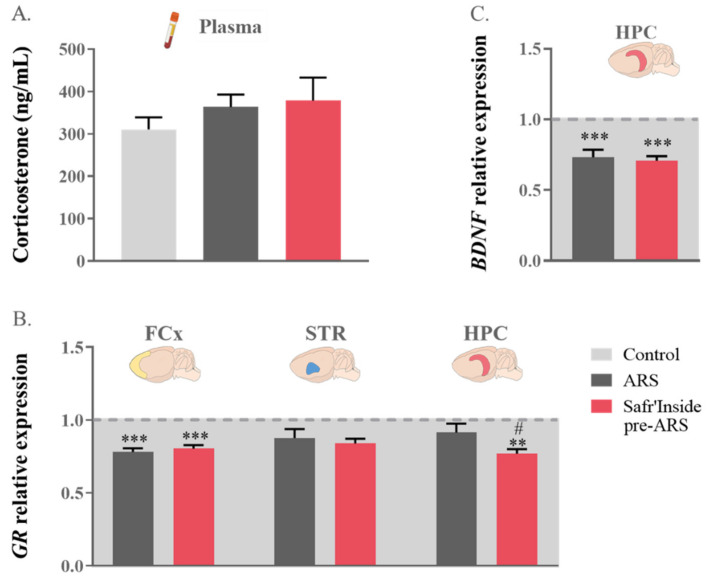
Effect of acute restraint stress (ARS) and oral pre-administration of Safr’Inside™ (6.25 mg/kg) on the hypothalamo-pituitary-adrenal (HPA) axis and hippocampal brain derived neurotrophic factor (BDNF) expression. (**A**) Plasma corticosterone levels expressed in ng/mL; (**B**) Glucocorticoid Receptor (GR) relative gene expression in the Frontal Cortex (FCx), Striatum (STR) and Hippocampus (HPC) and (**C**) HPC BDNF expression. For (**B**,**C**), data are represented as the foldchange calculated relative to the control group (baseline = 1). Results are shown as mean ± SEM. Control: *n* = 9–14; ARS: *n* = 10–15; Safr’Inside pre-ARS: *n* = 9–15. ** *p* ≤ 0.01, *** *p* ≤ 0.001 vs. Control; # *p* ≤ 0.05 vs. ARS.

In order to identify the neurobiological mechanisms potentially underlying the behavioral improvement induced by saffron extract when administered before ARS exposure, we assessed the impact of this treatment condition on stress-related neurobiochemical changes, starting with one of the main mediators of stress, the HPA axis. As shown in Figure 4A, circulating corticosterone levels measured just after the FST were similar in the Control, ARS, and Safr’Inside pre-ARS groups. Nevertheless, these groups differ regarding *GR* gene expression in the FCx (F_(1,41)_ = 4165; *p* ≤ 0.001) and the HPC (F_(1,36)_ = 864; *p* ≤ 0.05), but not the STR (Figure 4B). As revealed by the post hoc analysis, this expression was indeed significantly decreased by ARS in the FCx (ARS vs. Control: *p* ≤ 0.001) regardless of saffron extract administration (Safr’Inside pre-ARS vs. Control: *p* ≤ 0.001), while only pretreated stressed mice display reduced *GR* expression in the HPC (Safr’Inside pre-ARS vs. Control: *p* ≤ 0.01; Safr’Inside pre-ARS vs. ARS: *p* ≤ 0.05).

Since the behavioral effects of stress have been previously related to its negative impact on HPC neurogenesis, notably through corticosterone-induced impairment of *BDNF* expression [16], this was measured in the different experimental conditions. The one-way ANOVA analysis showed a difference in hippocampal *BDNF* gene expression among groups (F_(1,37)_ = 855; *p* ≤ 0.001; Figure 4C). As expected, exposure to ARS downregulates *BDNF* transcripts levels (ARS vs. Control: *p* ≤ 0.001). However, this downregulation was not changed by the pre-administration of Safr’Inside (Safr’Inside pre-ARS vs. Control: *p* ≤ 0.001), suggesting that its behavioral impact is unlikely related to a reduction of the deleterious effect of stress on hippocampal neurogenesis, at least as assessed through the local gene expression of *BDNF*.

### 3.4. Safr’Inside Administration Positively Regulates the Kynurenine Pathway

**Figure 5:**
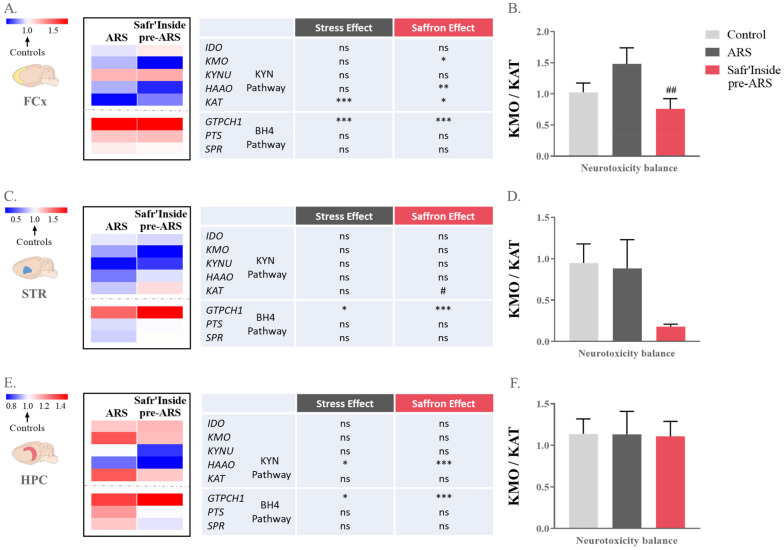
Effect of acute restraint stress (ARS) and oral pre-administration of Safr’Inside™ (6.25 mg/kg) on kynurenine and tetrahydrobiopterin (BH4) pathways. Heatmaps showing expression levels (as compared to control) of key enzymes of KYN and BH4 pathway in (**A**) the Frontal Cortex (FCx); (**C**) the Striatum (STR); (**E**) the Hippocampus (HPC); and KMO/KAT ratio representing KYN-related neurotoxicity balance in (**B**) the FCx; (**D**) the STR; (**F**) the HPC. Control: *n* = 5–14; ARS: *n* = 5–15; Safr’Inside pre-ARS: *n* = 5–15. * *p* ≤ 0.05, ** *p* ≤ 0.01, *** *p* ≤ 0.001 vs. Control; # *p* ≤ 0.05, ## *p* ≤ 0.01 vs. ARS. IDO: Indoleamine 2,3-Dioxygenase; KMO: Kynurenine 3-Monooxygenase; KYNU: Kynureninase; HAAO: 3-Hydroxyanthranilate 3,4-Dioxygenase; KAT: Kynurenine Aminotransferase; GTPCH1: GTP-Cyclohydrolase I; PTS: 6-Pyruvoyl Tetrahydropterin synthase; SPR: Sepiapterin Reductase.

Several studies report that activation of the KYN pathway, which is known to contribute to inflammation-related depressive-like behavior [20,22,55], is also found in different stress models of depression [45,46,56,57]. In line with these findings, we assessed KYN pathway activation by measuring brain expression levels of its key enzymes. The statistical analysis revealed differential effects of ARS and treatment depending on the enzyme and brain area considered. In the FCx, we observed significant differences between groups regarding the expression of two important enzymes of the neurotoxic side of the KYN pathway, namely *KMO* (F_(1,20)_ = 181; *p* ≤ 0.05) and HAAO (Kruskal-Wallis analysis: *p* ≤ 0.05; Figure 5A). Interestingly, they are both decreased by Safr’Inside administration as compared to controls (Safr’Inside pre-ARS vs. Control; *KMO: p* ≤ 0.05 and *HAAO: p* ≤ 0.01). Additionally, the expression of *KAT*, the enzyme conversely promoting neuroprotection, is also changed in this brain area (F_(1,15)_ = 385; *p* ≤ 0.01). Specifically, ARS drastically downregulates *KAT* expression (ARS vs. Control: *p* ≤ 0.001), but this effect is partially blunted by Safr’Inside administration (Safr’Inside pre-ARS vs. Control: *p* ≤ 0.05). Akin to these findings, the neurotoxicity ratio, as reflected by the *KMO*/*KAT* ratio, is also different depending on the group considered (F_(1,13)_ = 96,3; *p* ≤ 0.05; Figure 5B). The post hoc analysis showed that this ratio is significantly lower in mice pretreated with Safr’Inside than in untreated stressed mice (Safr’Inside pre-ARS vs. ARS: *p* ≤ 0.01). In the STR, *KAT* was also differentially expressed among groups (Kruskal–Wallis analysis: *p* ≤ 0.05; Figure 5C), this expression being significantly higher in saffron-treated mice than in untreated-stressed group (Safr’Inside pre-ARS vs. ARS: *p* ≤ 0.05). Accordingly, the neurotoxicity ratio tends to be reduced by Safr’Inside pretreatment (Kruskal–Wallis analysis: *p* = 0.06; Figure 5D). In the HPC, this ratio was similar in the different groups. However, the one-way ANOVA showed a significant effect on the hippocampal expression of *HAAO* (F_(1,36)_ = 960; *p* ≤ 0.01; Figure 5E). Indeed, ARS decreases *HAAO* expression (ARS vs. Control: *p* ≤ 0.05), with this reduction being even stronger in Safr’Inside-treated mice (Safr’Inside pre-ARS vs. Control: *p* ≤ 0.001). Overall, these data suggest that saffron extract administration reduces KYN-related neurotoxicity in a brain area dependent manner.

Together with the KYN pathway, the BH4 pathway, whose activity is changed by immobilization stress [58], also participates to the induction of depressive symptoms [22]. Therefore, it may similarly play a role in Safr’Inside-induced behavioral improvement. This does not seem however to be the case, as revealed by assessment of gene expression of several key elements of the BH4 pathway, including GTPCH1, the first and limiting enzyme of the pathway that, together with PTS and SPR, leads to BH4 synthesis [22,23]. Indeed, although *GTPCH1* expression is significantly altered by the experimental conditions in the three brain areas of interest (FCx: F_(1,40)_ = 438; *p* ≤ 0.001; STR: F_(1,25)_ = 282; *p* ≤ 0.01; HPC: Kruskal–Wallis analysis *p* ≤ 0.001; Figure 5A,C,E respectively), the post hoc analyses revealed that ARS increases *GTPCH1* expression regardless of saffron administration (FCx: ARS vs. Control: *p* ≤ 0.001; Safr’Inside pre-ARS vs. Control: *p* ≤ 0.001; STR: ARS vs. Control: *p* ≤ 0.05; Safr’Inside pre-ARS vs. Control: *p* ≤ 0.001; and HPC: ARS vs. Control: *p* ≤ 0.05; Safr’Inside pre-ARS vs. Control: *p* ≤ 0.001). In addition, gene expression of *PTS* and *SPR* is unchanged whatever the brain area and the experimental condition.

### 3.5. Safr’Inside Administration Partially Prevents ARS-Induced Alterations of Neurotransmission

**Figure 6:**
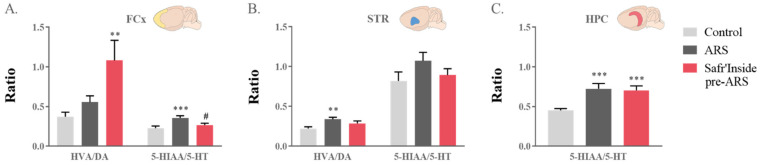
Effect of acute restraint stress (ARS) and oral pre-administration of Safr’Inside™ (6.25 mg/kg) on monoamine catabolism. HVA/DA and 5-HIAA/5-HT ratios reflect the catabolism of DA and 5-HT respectively. Ratios in (**A**) the Frontal Cortex (FCx); (**B**) Striatum (STR); (**C**) Hippocampus (HPC). Results are shown as mean ± SEM. Control: *n* = 12–14; ARS: *n* = 12–15; Safr’Inside pre-ARS: *n* = 13–15. ** *p* ≤ 0.01, *** *p* ≤ 0.001 vs. Control; # *p* ≤ 0.05 vs. ARS. DA: dopamine; HVA: homovanillic acid; 5-HT: serotonin; 5-HIAA: 5-hydroxyindolacetic acid.

Based on the current knowledge of the mechanisms of action of conventional ADs and our recent data showing that Safr’Inside administration improves monoaminergic neurotransmission in basal conditions [38], we next measured its potential impact on ARS-induced monoamine alterations. For this purpose, we first assessed whole tissue contents of 5-HT, DA and their metabolites in the three structures of interest (Table 1). Neither ARS exposure nor administration of Safr’Inside significantly changed 5-HT and DA levels. However, they differentially alter their metabolite concentrations depending on the brain area, except for DOPAC whose levels, when detectable, were similar in all mice. Regarding 5-HIAA levels, statistical analyses revealed differences between groups in the FCx (Kruskal–Wallis analysis: *p* ≤ 0.01), STR (F_(1,24)_ = 253; *p* ≤ 0.01) and HPC (Kruskal–Wallis analysis: *p* ≤ 0.001). Indeed, ARS increases 5-HIAA concentrations in the three brain areas of untreated stressed mice (ARS vs. Control: FCx and HPC: *p* ≤ 0.001; STR: *p* ≤ 0.01), while the pre-administration of Safr’Inside only prevents this increase in the FCx (Safr’Inside pre-ARS vs. Control: STR and HPC: *p* ≤ 0.01). Consistent with this, 5-HT turnover ratio (5-HIAA/5-HT) was different between groups in the FCx (F_(1,40)_ = 349; *p* ≤ 0.01; Figure 6A) and HPC (Kruskal–Wallis analysis: *p* ≤ 0.001; Figure 6C). Indeed, ARS significantly augments this ratio in the FCx of untreated (ARS vs. Control: *p* ≤ 0.001; Figure 6A), but not treated, stressed mice (Safr’Inside pre-ARS vs. ARS: *p* ≤ 0.05). On the other hand, it was enhanced in the HPC of all stressed mice, regardless of saffron administration (ARS vs. Control: *p* ≤ 0.001; Safr’Inside pre-ARS vs. Control: *p* ≤ 0.001; Figure 6C).

The one-way ANOVA also showed increased levels of HVA, the final DA metabolite, in the FCx (F_(1,38)_ = 188; *p* ≤ 0.05) and the STR (Kruskal–Wallis analysis: *p* ≤ 0.05; Table 1). In the FCx, this is related to an ARS effect independent from treatment (ARS vs. Control: *p* ≤ 0.01), although the local DA turnover ratio (HVA/DA) is enhanced in saffron-treated mice (Kruskal–Wallis analysis: *p* ≤ 0.01; Safr’Inside pre-ARS vs. Control: *p* ≤ 0.01; Figure 6A). On the contrary, the ARS-induced enhancement of HVA levels reported in the STR of untreated stressed mice (ARS vs. Control: *p* ≤ 0.05, Table 1) is abolished by Safr’Inside pretreatment, as revealed by the multiple group analysis. Consequently, the striatal DA turnover ratio is significantly increased in stressed mice (Kruskal–Wallis analysis: *p* ≤ 0.05; ARS vs. Control: *p* ≤ 0.01; Figure 6B), unless they were pretreated with Safr’Inside.

### 3.6. Safr’Inside Administration Modulates Key Elements of Monoaminergic Systems

In order to further study the impact of Safr’Inside on ARS-induced alterations of brain monoamine systems, we measured gene expression and/or protein level of several of their key regulatory elements [9,11,59,60,61,62,63,64]. This includes different receptors (5-HTR1a; 5-HTR1b; DRD1; DRD2), transporters (SERT; DAT), and degradation enzymes (MAO-A, particularly targeting 5-HT; COMT, selectively catabolizing DA; and MAO-B that metabolizes the two monoamines, but with a much higher affinity for DA).

**Figure 7:**
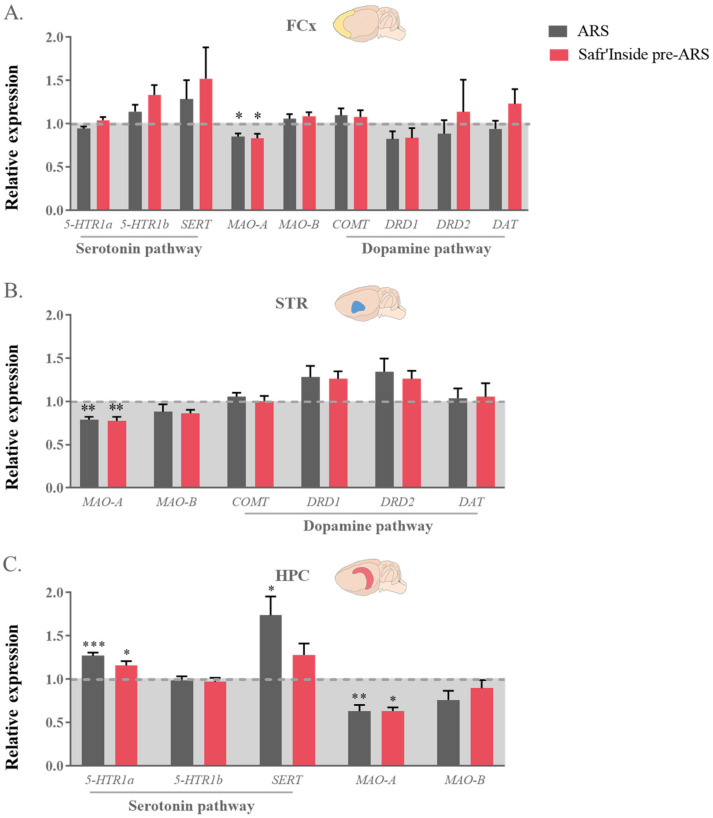
Effect of acute restraint stress (ARS) and oral pre-administration of Safr’Inside™ (6.25 mg/kg) on monoamine receptors, transporters and enzymes genes expression. Genes expression in (**A**) the Frontal Cortex (FCx); (**B**) Striatum (STR) and (**C**) Hippocampus (HPC). Data are represented as the foldchange calculated relative to the control group (baseline = 1). Results are shown as mean ± SEM. Control: *n* = 6–14; ARS: *n* = 9–15; Safr’Inside pre-ARS: *n* = 8–15. * *p* ≤ 0.05, ** *p* ≤ 0.01, *** *p* ≤ 0.001 vs. Control. 5-HTR1a and b: Serotonin 1a and 1b Receptors; SERT: Serotonin transporter; MAO-A and B: Monoamine oxidase A and B; COMT: Catechol-O-methyltransferase; DRD1 and DRD2: Dopamine Receptors D1 and D2; DAT: Dopamine transporter.

**Figure 8:**
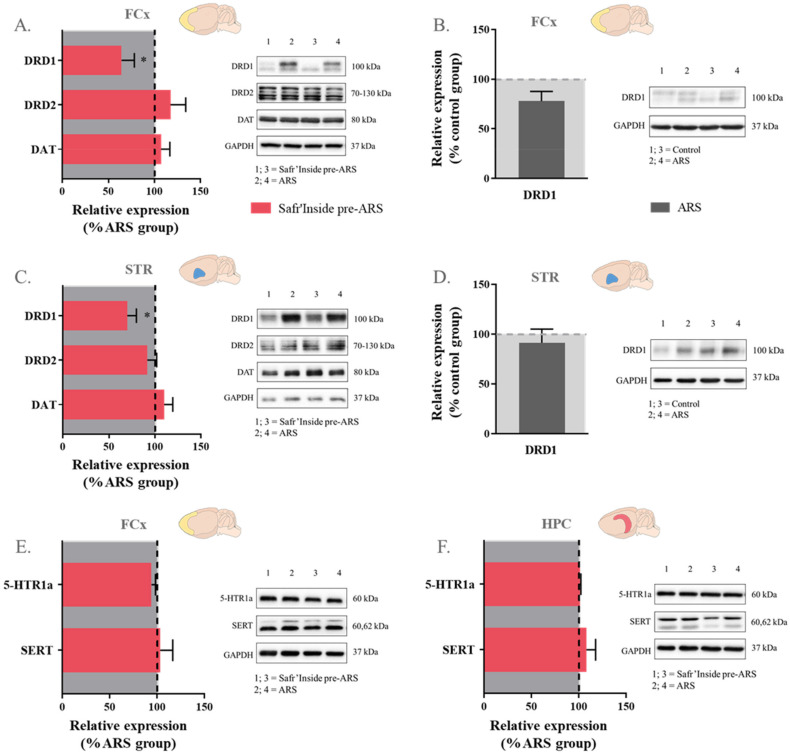
Effect of acute restraint stress (ARS) and oral pre-administration of Safr’Inside™ (6.25 mg/kg) on protein expression of monoamine receptors and transporters. (**A**) Effect of Safr’Inside compared to ARS group on DA system proteins in the Frontal Cortex (FCx); (**B**) Effect of ARS compared to control group on DRD1 protein in the FCx; (**C**) Effect of Safr’Inside compared to ARS group on DA system proteins in the Striatum (STR); (**D**) Effect of ARS compared to control group on DRD1 proteins in the STR; (**E**) Effect of Safr’Inside compared to ARS group on 5-HT system proteins in the FCx; (**F**) Effect of Safr’Inside compared to ARS group on 5-HT system proteins in the Hippocampus (HPC). Results are shown as mean ± SEM. Control: *n* = 13–14; ARS: *n* = 11–14; Safr’Inside pre-ARS: *n* = 12–14. * *p* ≤ 0.05. DRD1 and DRD2: Dopamine Receptors D1 and D2; DAT: Dopamine transporter; 5-HTR1a: Serotonin 1a Receptor; SERT: Serotonin transporter.

Regarding the 5-HT pathway, statistical analyses revealed differences between groups in *MAO-A* expression in the FCx (F_(1,40)_ = 1226; *p* ≤ 0.05; Figure 7A), STR (F_(1,24)_ = 962; *p* ≤ 0.01; Figure 7B) and HPC (Kruskal–Wallis analysis: *p* ≤ 0.01; Figure 7C). As compared to controls, this expression is indeed decreased in stressed mice, whether they are treated (Safr’Inside pre-ARS vs. Control: *p* ≤ 0.05 in the FCx and HPC and *p* ≤ 0.01 in the STR) or not with saffron (ARS vs. Control: *p* ≤ 0.05 in the FCx and HPC and *p* ≤ 0.01 in the STR). Concerning 5-HT receptors and transporter, no differences were observed at the protein level in the FCx and HPC (Figure 8E,F), but their gene expression does change among groups in the HPC (F_(1,37)_ = 1635; *p* ≤ 0.01 and Kruskal-Wallis analysis: *p* ≤ 0.05 for 5-HTR1a and SERT respectively; Figure 7C), a particularly important brain area for the therapeutic effect of serotoninergic ADs [65,66]. Specifically, ARS increases the expression of *5-HTR1a* in all stressed mice (ARS vs. Control: *p* ≤ 0.001; Safr’Inside pre-ARS vs. Control: *p* ≤ 0.05; Figure 7C), while that of *SERT* is only upregulated by ARS in the absence of saffron pretreatment (ARS vs. Control; *p* ≤ 0.05; Figure 7C), which prevents this effect.

Concerning the DA pathway, no difference between groups was reported whatever the brain area for the gene expression of the enzymes more specifically involved in DA catabolism (*COMT* and *MAO-B*), as well as dopaminergic receptors and transporter (Figure 7), meaning that neither ARS nor saffron administration change the expression of these factors. These results were confirmed at the protein level for DRD2 and DAT (Figure 8), but differ regarding DRD1. Indeed, Safr’Inside-treated stressed mice display decreased DRD1 protein levels in the FCx (t_(1,22)_ = 2.14; *p* ≤ 0.05; Figure 8A) and STR (Mann–Whitney U test: *p* ≤ 0.05; Figure 8C) compared to untreated stressed mice, although ARS does not significantly change these levels (Figure 8B,C).

**Figure 9:**
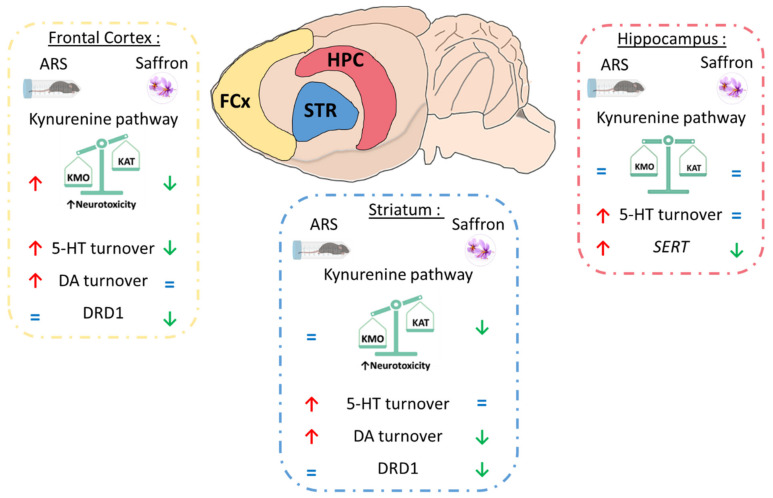
Summary of the effects of saffron on acute restraint stress (ARS)-induced neurobiological changes depending on the brain area. KMO: kynurenine 3-monooxygenase; KAT: kynurenine aminotransferase; 5-HT: serotonin; DA: dopamine; DRD1: Dopamine Receptor D1; SERT; serotonin transporter.

Taken together, these results show that Safr’Inside administration modulates KYN pathway activation, as well as dopaminergic and serotonergic neurotransmission, which may contribute to its preventive effect on ARS-induced depressive-like behavior (Figure 9).

## 4. Discussion

Due to the high failure rate and associated side effects of classical ADs, more and more studies search for natural alternatives to improve the management of mood disorders. If promising results are increasingly reported regarding saffron supplementations, the underlying mechanisms remain largely misunderstood. Here, we show for the first time that saffron extract interferes with ARS-induced depressive-like behavior when administered before, but not after, stress exposure. Importantly, we also report that Safr’Inside pretreatment concomitantly reduces KYN-related neurotoxicity and improves stress-induced monoamine system dysregulation in a brain area-dependent manner. Hence, this study highlights the ability of saffron extracts to improve depressive-like behavior under stress conditions, which are recognized predictors of depression, while finely regulating the function of key systems in the pathophysiology of the disease.

The ARS is a well validated paradigm to study stress-induced depressive-like behavior, since it causes several emotional and neurobiological alterations modeling those reported in depressive disorders [45,46,47,67]. Accordingly, untreated mice submitted to ARS in the current study displayed increased depressive-like behavior, as particularly shown by the high proportion of mice from this group spending significantly more time immobile than controls in the FST, which is widely used to preclinically test candidate compounds for their antidepressant activity [68]. As expected, this is associated with HPA axis dysregulation and alterations of neurogenesis, KYN pathway activation, and monoamine neurotransmission. Exposing saffron-treated mice to the ARS procedure allowed testing whether the antidepressant-like properties of saffron previously reported in unstimulated conditions (i.e., unstressed mice) [34,35,38] extend to stress conditions, as recently reported for some of its bioactive compounds [68,69]. Interestingly, several lines of compelling evidence strongly suggest that this is the case. Indeed, we showed here that administrating Safr’Inside before ARS onset normalized the proportion of mice being highly immobile, which was doubled in untreated stressed group compared to the control group. Consistent with this, stress-induced increase of immobility was not detected in mice pretreated with saffron, which behaved as control mice in the FST, therefore supporting the fact that saffron is effective in reducing stress-induced depressive-like behavior. It could be argued that saffron pretreated mice were not significantly different from untreated stressed mice either, which might suggest that the lack of increased immobility might instead simply reflect a non-specific response. However, this is unlikely, since saffron pretreatment also concomitantly targeted the neurobiological processes known to underly the reported behavioral alterations. Moreover, the behavioral effect of stress remained significant in mice treated after stress, whereas the two saffron-treated groups only differed by the time of saffron administration. In addition, the current results fit with compelling clinical and preclinical studies reporting its ability to improve mood and depressive symptoms [26,31,34,35,70,71], including in stressful conditions, although the number of studies is much less in this case [68,69,72]. Taken together, these findings highlight the need of investigating further the behavioral impact of saffron under stress conditions. Meanwhile, the current study already provides new and valuable information on the antidepressant-like properties of saffron in that context. Importantly, it shows that a behavioral effect was detected despite the very low dose used here (6.25 mg/kg *per os*), as compared to those reported in the literature [34,35,71,73]. It is worth mentioning that this dose was initially calculated based on that classically administered to humans (30 mg/day) by using the guidelines for dose-equivalence calculation provided by the FDA [74]. Altogether, these findings support the translational relevance of the present study.

Since the HPA axis is one of the main mediators of stress and the first to be activated upon stress exposure, it may appear as a likely target of saffron to drive its behavioral impact. In line with this assumption, a few studies previously reported that saffron reverses stress-induced increase in corticosterone levels [42,44], but this is not always the case [38]. Here, we cannot definitively conclude about the potential effect of Safr’Inside administration on stress-induced increase in corticosterone levels, since this increase, although expected based on other studies [45,46,47], was not detected in the current experiment. It is noteworthy, however, that corticosterone levels were measured almost 5 h after ARS onset and in blood samples collected right after the FST, which may have stressed control mice, as suggested by their corticosterone levels. Measuring corticosterone at different time points during ARS exposure and/or right at its end, rather than after the FST, should help address this issue. It was however not possible to carry out this time-course in the present study. Meanwhile, the fact that saffron extract pretreatment does not reverse the ARS-induced decrease of *GR* expression that is reported in the FCx in agreement with previously published data [45,46], argues against a main role of HPA axis modulation by Safr’Inside in its protective behavioral effect. Similarly, it does not seem to act by reducing the impact of stress on hippocampal neurogenesis, at least as assessed through the expression of BDNF, which is one of the main intermediates between the impairment of stress-induced hippocampal neurogenesis and development of related depressive symptoms [16,18,75]. In addition, the gene expression of this important neurotrophic factor is well-known to be under GR-mediated regulation in stressed conditions [76]. Consistent with the literature [45,46], we show here that ARS decreases hippocampal *BDNF* expression. Importantly, this down-regulation is not prevented by saffron extract, despite its protective effect against ARS-induced depressive-like behavior. Of note, however, if these data argue against a main role of BDNF, they do not discard the involvement of other neurotrophic factors. Supporting this, different saffron extracts have been recently shown to upregulate protein and transcripts levels of several neurotrophic factors, including BDNF, although this was reported in other experimental conditions and after chronic administration [73,77,78]. On the other hand, BDNF hippocampal alterations related to depression seem to be preferentially associated with cognitive rather than emotional symptom dimensions [79]. Together, these findings highlight the need to deeply study the impact of saffron extracts on neurogenesis, particularly by considering other neurotrophic factors and behavioral endpoints, but this is beyond the scope of the present study.

Mounting evidence points to inflammation-driven alterations of the KYN and BH4 pathways as key players in the induction of depressive symptoms reported in inflammatory and/or stress conditions, due to their overall impact on 5-HT and DA metabolism, as well as increased oxidative damages and glutamate-related neurotoxicity [20,22,55,58]. Accordingly, they are increasingly considered as potential targets for the development of new therapeutic strategies in those conditions [22,80]. In agreement with previously published data [45,46,56,57,58], the two pathways are altered by ARS in untreated mice. However, we show for the first time that these alterations are differentially impacted by saffron. Increased BH4 synthesis resulting from upregulation of GTPCH1 activity has been previously shown to play a key role in ARS-induced oxidative damages [58]. We did not assess BH4 levels nor indices of oxidative stress, but our data on the impact of ARS on the BH4 pathway suggest that the same could likely happen here. However, this assumption, as well as the potential link between these alterations and increased depressive-like behavior have yet to be demonstrated. Meanwhile, the fact that saffron pretreatment did not reduce ARS-induced changes of the BH4 pathway suggests that it unlikely mediates behavioral improvement. On the contrary, we report that saffron targets different KYN enzymes depending on the brain area, which could in turn contribute to reducing the imbalance between the neuroprotective and neurotoxic sides of the KYN pathway, as suggested by calculation of the neurotoxicity ratio. Indeed, saffron decreases the expression of enzymes promoting oxidative stress and glutamate-related neurotoxicity in the FCx, and rather increases *KAT* expression in the STR, therefore favoring the local synthesis of the neuroprotective KYN metabolite, kynurenic acid (KYNA). It could be argued that changes of gene expression do not necessarily imply concomitant changes of enzymatic activity. However, several studies previously reported that it is actually the case for KYN pathway enzymes [55,81]. Taken together, our results suggest that Safr’Inside-induced modulation of KYN-related glutamate-neurotoxicity may contribute to reduce associated depressive-like behavior. This assumption fits with mounting studies highlighting the link between generation of neurotoxic KYN metabolites, particularly quinolinic acid (QUIN) that promotes excitotoxicity by binding to NMDA glutamatergic receptors, and the severity of depressive symptoms [22,80,82]. It is also supported by preclinical studies reporting that different phytochemical compounds contained in Safr’Inside, particularly safranal and crocins, protect against brain oxidative damages induced by QUIN administration [83] and behavioral alterations associated with direct manipulations of NMDA receptor activation [84]. Interestingly, a recent study reports that counteracting QUIN effects by pharmacologically blocking these receptors with ketamine prevents the induction of depressive-like behaviors in a murine model of inflammation [85]. In line with this preclinical data, ketamine infusion in depressed patients resistant to ADs has been shown to improve their depressive symptomatology [85]. Moreover, their KYNA/QUIN ratio predicts their response to ketamine. These findings point to a modulation of KYN pathway-driven neurotoxicity as a promising new strategy of treatment and, together with the present study, arouse the interest of testing therapeutic approaches using saffron in this context.

The 5-HT system is a well-known target of stress and an important player in the etiology and treatment of depressive disorders [9,10,11,86]. Conversely, most conventional ADs aim to restore serotonergic neurotransmission, mainly by acting on 5-HT reuptake or catabolism [12,87]. Here, ARS increases *SERT* expression in the HPC and 5-HIAA concentrations in all brain areas assessed. It also increases 5-HT turnover, as assessed through the 5-HIAA/5-HT ratio, in the FCx and HPC. These results agree with previous studies using other stress paradigms [88,89,90,91,92,93]. ARS also downregulates *MAO-A* expression, the enzyme responsible for 5-HT degradation. Although this result may appear as counter-intuitive, it is consistent with previously published data [76]. In addition, it is worth mentioning that the impact of acute stress on *MAO-A* expression has been shown to change over time, as for most GR-responsive genes [76]. Here, *MAO-A* expression and 5-HIAA levels were assessed only once and at the same time point, which was not necessarily suitable to see the causal link between the two measures. Similarly, the fact that we simultaneously assessed the impact of stress on the hippocampal gene expression of *5-HTR1a*, which is increased in stressed mice as previously shown [94], and its local protein levels, yet unchanged by that time, also likely explain this apparent discrepancy. Further studies would be required to obtain a dynamic overview of the effect of stress on each of these 5-HT factors and their potential interdependence, but this was not the question addressed in the present study. Importantly, we show that the pre-administration of saffron prevents ARS-induced impairment of 5-HT neurotransmission, including by acting on the same targets than conventional ADs [72]. Indeed, it blocks the increase of 5-HIAA levels and 5-HIAA/5-HT ratio in the FCx, as well as *SERT* upregulation in the HPC. This last result is consistent with our previously published data [38] and particularly interesting in light of the key role of the HPC in the therapeutic properties of serotonergic ADs [65,66]. Also in agreement with our earlier study [38], saffron extract does not change the hippocampal *5-HTR1a* expression, whose increase by ARS would be more related to an adaptive response to stress than to the behavioral alterations it elicits [95]. Altogether, these results point to saffron-induced modulation of 5-HT neurotransmission as an important player in the associated improvement of depressive-like behavior.

Along with the serotonergic system, the dopaminergic mesolimbic and mesocortical pathways are altered upon stress exposure, as well as in depression [11,12,96,97]. Specifically, DA oxidation products that result from its increased turnover and catabolism have been suggested to contribute to the pathophysiology of neuropsychiatric disorders and stress-induced depression [93]. In accordance with findings reporting high HVA concentrations in rodents exposed to acute stress [98], here we show that ARS significantly increases the levels of this metabolite in the FCx and STR, although with the values being much higher in this last brain area, suggesting local enhancement of DA turnover. Supporting this assumption, the HVA/DA ratio is augmented by ARS in the STR, this increase not reaching significance in the FCx. Importantly, saffron extract pretreatment prevents the effect of ARS on both HVA levels and HVA/DA ratio in the STR, but not the FCx. Of note, this ratio is even enhanced a little more in the FCx of saffron treated mice, although with an important interindividual variability. The underlying reasons for this are not clear at this time, emphasizing the need to further study saffron-induced modulation of DA metabolism. Meanwhile, it is important to note that differential, or even opposite, modulations of the dopaminergic mesolimbic and mesocortical pathways have been already reported, including in the context of depression [99,100]. Actually, confirming the differential impact of saffron on DA neurotransmission according to the pathway should be particularly interesting considering their preferential involvement in different depressive symptoms, the mesolimbic pathway being for example particularly critical to those related to reward processing and motivation [12,100,101]. Interestingly, we also reported that DRD1 protein levels measured in the STR and FCx, two brain areas where this receptor is highly expressed [98], are lower in stressed mice receiving saffron than in their untreated counterparts. Akin to these data, a nutritional supplementation with n-3 polyunsaturated fatty acids, which are well-known for their beneficial effects on mood, has been recently shown to improve DA-related behavioral alterations by downregulating DRD1 levels [102]. It may be tempting to similarly propose a link between saffron-induced DRD1 downregulation and improvement of stress-induced depressive-like behavior. However, the lack of detectable impact of stress on DRD1 levels argues so far against this hypothesis. The significance of the impact of saffron on this receptor has yet to be elucidated, as well as its potential effect on other key markers of dopaminergic activity.

Overall, the present results already point to saffron-induced modulation of dopaminergic neurotransmission, together with serotonergic neurotransmission and activity of the kynurenine pathway, as likely mediators to improve stress-induced depressive-like behaviors. A limitation to this work is that the potential links between these different neurobiological systems cannot be deduced from the present findings. This study represents an essential first step, and upcoming experiments should overcome this limitation. The broad screening of the neurobiological effects of saffron carried out here will serve to refine the avenues to be explored regarding the mechanisms of action of saffron in the context of depressive disorders, as well as the symptoms to preferentially study in humans and/or to experimentally model in rodents.

## 5. Conclusions

In conclusion, this study provides valuable information on the protective impact of saffron against the development of depressive symptoms related to stress exposure. It also provides important cues on how to use saffron-based alternative nutritional strategies, considering the preventive treatment as a first-line solution to tackle the induction of depressive symptoms in that context. In addition, by highlighting the ability of saffron to differentially target several pathophysiological bases of depression, this work may provide insights concerning the symptom dimensions for which nutritional interventions with saffron might be effective, and by extension, the clinical profile of patients who may benefit from these alternative therapeutic approaches. Altogether, these findings represent useful information for the better management and treatment of depressive disorders with saffron supplementation.

## Figures and Tables

**Table 1:** Effect of acute restraint stress (ARS) and oral pre-administration of Safr’Inside™ (6.25 mg/kg) on monoamine levels and their metabolites measured by High Performance Liquid Chromatography (HPLC).

	FCx (nmoles/g)			STR (nmoles/g)			HPC (pmoles/g)		
	Control	ARS	Safr’Inside	Control	ARS	Safr’Inside	Control	ARS	Safr’Inside
[DA]	4.3 ± 1.1	4.1 ± 0.5	3.7 ± 1.0	49 ± 5.8	47.2 ± 3.4	42.9 ± 4.1	144 ± 33	125 ± 18	93 ± 15
[DOPAC]	1.2 ± 0.2	1.4 ± 0.2	0.95 ± 0.1	11.1 ± 1.2	9.5 ± 1	7.7 ± 0.7	n.d	n.d	n.d
[HVA]	1.1 ± 0.2	1.9 ± 0.2 **	1.7 ± 0.2 *	11 ± 1.3	15.8 ± 1.5 *	11.3 ± 1.1	n.d	n.d	n.d
[5-HT]	1.2 ± 0.05	1.4 ± 0.08	1.3 ± 0.7	0.75 ± 0.1	0.84 ± 0.09	0.97 ± 0.1	986 ± 121	1082 ± 134	938 ± 119
[5-HIAA]	0.27 ± 0.03	0.49 ± 0.04 ***	0.37 ± 0.04	0.52 ± 0.06	0.88 ± 0.1 **	0.83 ± 0.08 ^**^	401 ± 38	698 ± 55 ***	593 ± 43 **

The levels of dopamine (DA), 3,4-dihydroxyphenylacetic acid (DOPAC), homovanillic acid (HVA), serotonin (5-HT) and 5-hydroxyindolacetic acid (5-HIAA) are expressed in nmoles/g of tissue in the Frontal Cortex (FCx) and Striatum (STR), and pmoles/g in the Hippocampus (HPC). Results are shown as mean ± SEM. n.d: not detectable. Control: *n* = 11–14; ARS: *n* = 13–15; Safr’Inside pre-ARS: *n* = 13–15. * *p* ≤ 0.05, ** *p* ≤ 0.01, *** *p* ≤ 0.001 vs. Control.

## Data Availability

The dataset for this study is available on request from the corresponding author.

## Supplementary Materials

The following are available online at https://www.mdpi.com/article/10.3390/pharmaceutics13122155/s1, Table S1: Classification of the genes of interest and their references according to the systems or pathways they belong to, Table S2: Parameters used for each WB (sample concentration, saturation conditions, dilution of Ir antibody and reference of the appropriate IIr antibody).
