# Supplementary material for: Prevention of Stress-Induced Depressive-like Behavior by Saffron Extract Is Associated with Modulation of Kynurenine Pathway and Monoamine Neurotransmission
Source: Pharmaceutics. 2021 Dec 14;13(12):2155. doi: 10.3390/pharmaceutics13122155 (PMC8709346; doi:10.3390/pharmaceutics13122155)
Supplement: Supplementary file 1 [file pharmaceutics-13-02155-s001.zip › pharmaceutics-1489042-supplementary.pdf]

pharmaceutics

MDPI

Supplementary Materials: Prevention of Stress-Induced Depressive-Like Behavior by Saffron Extract is Associated with Modulation of Kynurenine Pathway and Monoamine Neurotransmission

Camille Monchaux De Oliveira, Véronique De Smedt-Peyrusse, Jennifer Morael, Sylvie Vancassel, Lucile Capuron, David Gaudout, Line Pourtau and Nathalie Castanon

Table S1. Classification of the genes of interest and their references according to the systems or pathways they belong to.

Category	Genes	Reference
Housekeeping gene	*Beta-2-Microglobulin (B2M)*	Mm00437762_m1
HPA axis	*Glucocorticoid Receptor (GR)*	Mm00433832_m1
Neurogenesis	*Brain-Derived Neurotrophic Factor (BDNF)*	Mm01334047_m1
Monoamine receptors	*Dopamine Receptor D1 (DRD1)*	Mm01353211_m1
	Dopamine Receptor D2 (DRD2)	Mm00438545_m1
	Serotonin 1a Receptor (5-HTR1a)	Mm00434106_s1
	Serotonin 1b Receptor (5-HTR1b)	Mm00439377_s1
Monoamine degradation enzymes	*Monoamine Oxidase A (MA0-A)*	Mm00558004_m1
	Monoamine Oxidase B (MAO-B)	Mm00555412_m1
	Catechol-O-methyltransferase (COMT)	Mm00514377_m1
Monoamine transporters	*Dopamine transporter (DAT)*	Mm00438388_m1
	Serotonin transporter (SERT)	Mm00439391_m1
Kynurenine pathway	*Indoleamine 2,3-Dioxygenase (IDO)*	Mm00492590_m1
	Kynurenine 3-Monooxygenase (KMO)	Mm00505511_m1
	Kynureninase (KYNU)	Mm00551012_m1
	3-Hydroxyanthranilate 3,4-Dioxygenase (HAAO)	Mm00517945_m1
	Kynurenine Aminotransferase (KAT)	Mm01351821_m1
BH4 pathway	*GTP-Cyclohydrolase I (GTPCH1)*	Mm01322973_m1
	6-Pyruvoyl Tetrahydropterin (PTS)	Mm00478494_m1
	Sepiapterin Reductase (SPR)	Mm00488430_m1

Table S2. Parameters used for each WB (sample concentration, saturation conditions, dilution of Ir antibody and reference of the appropriate IIr antibody).

	Protein Name	Sample Concentration	Saturation	Ir Antibody Concentration	IIr Antibody
FCx	DRD1	10 µg	Milk 5%	1/1000	α Rat (712-035-150/Jackson)
	DRD2	10 µg	Milk 5%	1/1000	α Rabbit (711-035-152/Jackson)
	DAT	10 µg	Milk 5%	1/2000	α Rabbit (711-035-152/Jackson)
	5-HTR1a	5 µg	Milk 5%	1/1000	α Rabbit (711-035-152/Jackson)

	SERT	10 μg	BSA 5%	1/1000	α Rabbit (711-035-152/Jackson)
STR	DRD1	5 μg	Milk 5%	1/1000	α Rat (712-035-150/Jackson)
	DRD2	5 μg	Milk 5%	1/1000	α Rabbit (711-035-152/Jackson)
	DAT	5 μg	Milk 5%	1/2000	α Rabbit (711-035-152/Jackson)
HPC	5-HTR1a	5 μg	Milk 5%	1/1000	α Rabbit (711-035-152/Jackson)
	SERT	10 μg	BSA 5%	1/1000	α Rabbit (711-035-152/Jackson)
	GAPDH	-	Milk 5%	1/20000 or 25000	α Rabbit (711-035-152/Jackson)
